# Homœopathy in Michigan University

**Published:** 1873-07-15

**Authors:** 


### HOMŒOPATHY IN MICHIGAN UNIVERSITY.

At a late meeting, the Board of Regents of Michigan University passed the following resolutions:

WHEREAS, The Legislature of the State of Michigan, at its last session, re-enacted the law of 1855, requiring the appointment of Homœopathic Professors in the Medical Department of the University; and, whereas, it has always been claimed by the Board of Regents that the law was an infringement upon the rights and prerogatives of the Board; and, whereas, the Supreme Court of the State has refused to grant a *mandamus* requiring the Regents to comply with the law, thereby substantially confirming their action, therefore,

*Resolved*, That we maintain the position heretofore taken, and decline to make the appointments required by the law.

*Resolved further*, That we do this in no spirit of factious opposition to the apparent will of the Legislature, but because we believe the true and best interests of the University demand it.

*Resolved*, That we re-affirm the former action of the Board expressing a willingness to take official charge of an independent school of Homœopathy, and connect it with the University, whenever the means shall be provided for the payment of its professors.

